# Trunk muscle strength assessment as a predictor of complications in patients undergoing incisional hernia repair

**DOI:** 10.1007/s10029-026-03621-x

**Published:** 2026-03-21

**Authors:** P. Bravo-Ratón, J. C. Santos-Sánchez, J. L. Gil-Delgado, A. Sánchez-Arteaga, J. Tinoco-González, B. Sañudo Corrales, L. Tallón-Aguilar

**Affiliations:** 1 Department of Surgery, Abdominal Wall and Hernia Surgery Unit, Virgen del Rocio University Hospital, Avda. Manuel Siurot, S/N, 41013 Seville, Spain; 2 Department of Surgery, Medical Student, Virgen del Rocio University Hospital, Seville, Spain; 3 Department of Physical Education and Sport, University of Seville, Seville, Spain

**Keywords:** Incisional hernia, Trunk muscle strength, Abdominal wall, Postoperative complications, Prehabilitation

## Abstract

**Purpose:**

Incisional hernia is a common condition with significant complication rates. While various risk factors have been studied, the role of preoperative trunk muscle strength in predicting postoperative outcomes remains underexplored.

**Methods:**

This is a prospective cohort study conducted with 75 patients undergoing incisional hernia repair between 1st June 2022 to 31st September 2024. Preoperative trunk muscle strength was assessed by using a strain gauge device, targeting both medial and lateral abdominal muscle groups, and recording force peak (FP) and rate force development (RFD). Patients were followed for 30 days postoperatively, and complications were classified using the Clavien-Dindo classification. Associations between muscle strength and postoperative outcomes, anthropometric variables and comorbidities were analyzed.

**Results:**

Mean age was 57.6 years and mean BMI 29.19 kg/m^2^. Complications were observed in 32% of patients. Sex-related differences were found: men exhibited greater anterior trunk strength (FP90° 193.8 vs 147.4 N, *p* < 0.003; RFD90° 667 vs 400.9 N/s, *p* < 0.03). Postoperative bleeding was associated with lateral trunk strength (FP45° 96.0 vs 154.2 N; *p* < 0.042), and surgical site infection was correlated with anterior strength in male patients (FP90° 149.7 vs 203.8 N; *p* < 0.03). No significant association was found between bleeding and antiplatelet or anticoagulant therapy.

**Conclusion:**

Preoperative trunk muscle strength may serve as a useful predictor of postoperative complications in patients undergoing incisional hernia repair. These findings support the potential role of targeted pre-habilitation strategies, in order to improve surgical outcomes.

## Introduction

Incisional hernia (IH) remains a common complication following abdominal surgery, occurring in approximately 12.8% of patients within the first two years after a midline laparotomy [1]. Its overall incidence is heterogeneous and has varied over the past years, according to both surgical characteristics and patient’s comorbidities [2, 3]. Despite advances in surgical techniques, hernia recurrence rate ranges from 25–50% [4], and tend to increase with the number of prior hernia repairs, leading to greater morbidity and complication rates [5].

Several patient-related risk factors—including obesity, diabetes, immunosuppression or smoking [1, 3, 5–8]—are frequently and consistently associated with an increased postoperative morbidity and greater susceptibility to surgical site infection (SSI), which is the main risk factor for incisional hernia [5]. In spite of surgical techniques and mesh placement, preventing postoperative complications and recurrence remains a challenge [1–5, 9, 10]. While numerous studies have addressed anatomical and technical aspects of hernia repair, fewer have focused on the functional status of the abdominal wall prior to surgery [11, 12]. In particular, the role of preoperative trunk muscle strength as a predictor of surgical outcomes, has received limited attention, given its paramount importance by only scarce authors [13–15].

Emerging evidence suggests that trunk muscle function not only influences the biomechanics of hernia formation, but may also affect postoperative recovery and complication rates [11, 13–15]. Muscle strength has been proposed as a general indicator of overall health status [16–19], and several studies have reported associations between reduced abdominal wall strength and larger hernia defects [13–15]. In patients undergoing incisional hernia repair with European Hernia Society (EHS) classification grade ≥ W2, preoperative abdominal wall strength assessment has been shown to be feasible using both isokinetic dynamometry and portable isometric/strain-gauge techniques, with results suggesting that greater defect width may be associated with lower strength depending on the parameter assessed [13, 15, 21]. In addition, other studies have reported an inverse relationship between hernia size and abdominal wall muscle strength, demonstrating significant weakness in patients with large hernias (EHS grade W3, > 10 cm) [15]. Notably, they found that intraoperative measurements of hernia size were more strongly associated with strength deficits than preoperative CT imaging [15].

Despite the clinical relevance of these findings, most existing research relies on isokinetic dynamometry as the reference method for assessing muscle function. Although considered the gold standard, its routine use in clinical settings is limited due to high cost, bulky equipment, and limited accessibility [22–24].

In contrast, strain gauge systems provide a low-cost, portable, and reproducible alternative for the measurement of isometric muscle strength. While this technique has been validated in assessing various muscle groups—including ankle plantar flexors [21] and quadriceps [25]— its application in assessing abdominal muscle strength among patients suffering from abdominal wall hernias has not been extensively reported in the literature, despite the growing interest in functional evaluation of the abdominal wall in recent years [13–15].

It is widely recognized that the abdominal wall serves as a structural and functional unit, essential for maintaining core stability, respiratory mechanics, and intra-abdominal pressure [24, 26–28]. Experimental mechanobiology data suggest that reduced mechanical strain and altered load transmission may impair abdominal wall fibroblast function and collagen remodeling, potentially contributing to fascial weakness and suboptimal tissue repair [29, 30]. Accordingly, objective preoperative quantification of trunk muscle function may provide clinically relevant information for perioperative risk stratification and may inform future targeted prehabilitation strategies.

Therefore, the aim of this study was to evaluate the association between preoperative trunk muscle strength, assessed with a strain gauge, and the appearance of early postoperative complications, in patients undergoing incisional hernia repair. Furthermore, we studied the relationship between trunk muscle strength and both anthropometric and clinical variables, to assess its potential prognostic marker and support future prehabilitation strategies.

## Methods

### Study design, setting, ethics and registration

This prospective observational cohort study included adults undergoing elective incisional hernia repair at a tertiary-care hospital between June 2022 and September 2024. The study protocol was approved by the local ethics committee, and written informed consent was obtained from all participants prior to enrollment. This observational study was not prospectively registered.

### Participants

Inclusion criteria were: age ≥ 18 years, elective incisional hernia repair, availability of a preoperative computed tomography (CT) scan, and eligibility for preoperative trunk muscle strength assessment using a strain gauge. Exclusion criteria were: lateral hernias, hernia defects < 1 cm, life expectancy < 1 year, or refusal to participate. A total of 75 patients meeting eligibility criteria were included.

### Baseline variables

Demographic and clinical data were collected, including age, sex, body mass index (BMI), and fat and muscle mass percentages assessed by bioelectrical impedance analysis (Tanita-BIA, Model T55896, Tanita Corporation of America, Inc, Arlington Heights, IL). Comorbidities (hypertension, diabetes, cardiovascular and pulmonary disease), smoking status, anticoagulant/antiplatelet use, maximum axial hernia diameter (measured on preoperative CT), ASA class, and surgical technique were recorded. Frailty was not formally assessed using a validated instrument (e.g., handgrip strength or a clinical frailty scale); therefore, baseline vulnerability may not be fully captured by routinely collected clinical variables alone.

### Outcomes

The primary outcome was the occurrence of any postoperative complication within 30 days after surgery, graded according to the Clavien–Dindo classification. Secondary outcomes included SSI (superficial/deep), postoperative bleeding, reintervention, readmission, and length of stay.

Outcomes were assessed up to postoperative day 30 through outpatient visits and electronic medical record review; if in-person assessment was unavailable, patients were contacted by telephone using a standardized questionnaire. Follow-up at 30 days was complete in 75/75 patients (100%).

### Trunk muscle strength assessment

Trunk muscle strength was measured preoperatively using a strain gauge (Chronojump® Boscosystem, Barcelona, Spain). Patients were seated with hips and knees at 90° and wore a thoracic harness. Anterior (90° flexion) and lateral (45° rotation) isometric contractions were recorded in triplicate, using 5-s contractions and 60-s rest intervals. Force peak (FP, N) and rate of force development (RFD, N/s) were recorded at 90° and 45°. Prior to testing, a standardized warm-up and familiarization session was conducted to ensure consistent effort and adequate understanding of the procedure.

### Statistical analysis

Statistical analysis was performed using IBM SPSS Statistics for Windows, Version 24.0 (IBM Corp, Armonk, NY). Continuous variables are presented as mean ± standard deviation (SD) when approximately normally distributed, or as median (interquartile range, IQR) otherwise. Categorical variables are presented as n (%). Normality was assessed using the Shapiro–Wilk test. Between-group comparisons were performed using Student’s t-test or Mann–Whitney U test for continuous variables, and chi-square or Fisher’s exact test for categorical variables, as appropriate. Given the small and potentially selected laparoscopic subgroup, primary inferential analyses were restricted to open repairs. Correlations between maximum axial hernia diameter (continuous) and trunk strength parameters were assessed using Spearman’s rank correlation coefficient (two-sided), and correlations between age/BMI and strength parameters using Pearson’s correlation coefficient (two-sided), with pairwise deletion for missing data. A multivariable logistic regression model evaluated the association between preoperative PF45º (per 10 N) and any 30-day postoperative complication (composite outcome: wound complication, bleeding, reintervention, readmission and/or transfusion), adjusting for age, sex, BMI, ASA class (≥ III vs < III), and hernia diameter; adjusted odds ratios (aOR) with 95% confidence intervals (CI) are reported. The multivariable model used complete-case analysis. A two-sided α = 0.05 was applied; analyses were considered exploratory.

## Results

Given the small and potentially selected laparoscopic subgroup, inferential analyses (correlations and multivariable modelling) were restricted to open repairs; descriptive characteristics are reported for the overall cohort.

A total of 75 patients were included, all of whom underwent incisional hernia repair and had available preoperative trunk muscle strength measurements (medial and lateral abdominal muscles). The majority of patients were female (61.3%), with a mean age of 57.6 years (SD 11.54; IQR 52–66) and mean BMI of 29.19 kg/m^2^ (SD 5.31; IQR 25.05–33.19). Mean muscle mass was 60.9% (SD 10.67) and mean fat mass was 35% (SD 8.36). Hypertension was the most frequent comorbidity (36%), followed by diabetes and active smoking (both 25.3%). Most patients were ASA < III (68%). Mean maximum axial hernia diameter on CT was 5.85 cm (SD 2.64; IQR 4–7.5). Baseline and anthropometric characteristics are described in Table 1.

**Table 1:** Baseline and anthropometric characteristics

Sex (%)	Male: 29 (38.7%); Female: 46 (61.3%)	
Age (Mean ± SD) (IQR)	57.6 ± 11.54 years (52–66)	
BMI (Mean ± SD) (IQR)	29.19 ± 5.31 kg/m^2^ (25.05–33.19)	
%Muscle mass (Mean ± SD)	60.93 ± 10.67%	
%Fat mass (Mean ± SD)	35 ± 8.36%	
Smoking (%)	19 (25.3)	
Diabetes (%)	19 (25.3)	
Hypertension (%)	27 (36)	
Cardiovascular disease (%)	5 (6.7)	
Respiratory disease (%)	9 (12)	
Anticoagulant/antiplatelet (%)	7 (9.3)	
ASA (%)	< III 51 (68)	≥ III 24 (32)
Hernia diameter- CT Me ± SD (IQR)	5.85 ± 2.64 cm (4–7.5)	

Values are presented as mean ± standard deviation (SD) with interquartile range (IQR) in parentheses, unless otherwise specified. Categorical variables are shown as *n* (%). *Me* Mean; *SD* Standard deviation; *IQR* Inter-Quartile Range; *BMI* Body Mass Index; *ASA* American Society of Anesthesiologists; *CT* Computed Tomography

**Fig. 1:**
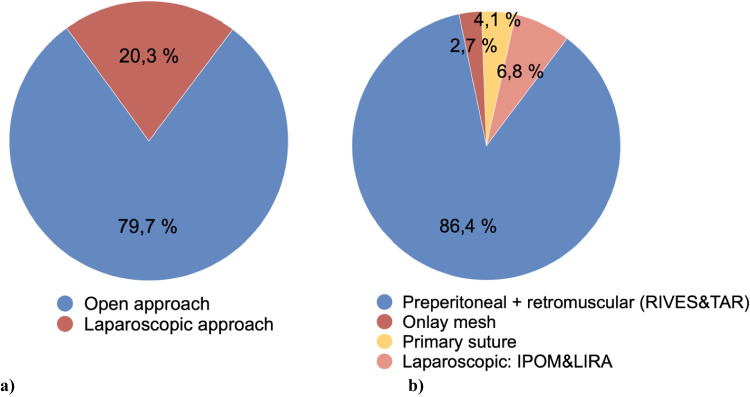
Surgical details and techniques. **a** Surgical approach distribution. **b** Distribution of surgical techniques according to the type of approach

Surgical details and techniques are shown in Table 2. Approach and technique data were available for 74 patients. An open approach was used in 79.7% of cases, while laparoscopic repair was used in 20.3% (Fig. 1). The most common technique was open retromuscular mesh placement (Rives–Stoppa) in 70.2% of patients, followed by open preperitoneal repair (12.2%). Transversus Abdominis Release (TAR) was employed in 4% of cases. Laparoscopic procedures were selected according to hernia location, prior surgical history and surgeon preference, with LIRA being the most frequently employed (4.1%), followed by IPOM (2.7%).

**Table 2:** Surgical details and techniques

Surgical Approach (*n* = 74)		
Open (%)	59 (79.7%)	
Laparoscopic (%)	15 (20.3%)	
Surgical Technique (*n* = 74)		
Preperitoneal repair	9 (12.2%)	
Retromuscular repair	RIVES-STOPPA technique 52 (70.2%)	TAR technique 3 (4%)
Preaponeurotic repair	2 (2.7%)	
IPOM	2 (2.7%)	
LIRA	3 (4.1%)	
Primary suture	3 (4.1%)	

Data available for *n* = 74; 1 missing. Percentages are valid percentages

Regarding trunk muscle function, mean FP90º (central musculature) was 164.78 ± 59.66 N, while FP45º (lateral musculature) was 151.15 ± 48.38 N. Mean RFD90º was 505.03 ± 443.23 N/s and mean RFD45º was 330.24 ± 265.04 N/s. An exploratory sex-stratified analysis for SSI is reported in Table 3.

**Table 3:** Association between trunk muscle strength and postoperative outcomes

	PF90 (*N*)	*p*	RFD90 (N/s)	*p*	PF45 (N)	*p*	RFD45 (N/s)	*p*
Bleeding								
*Yes*	157.21 (*n* = 4)	0.780	553.82 (*n* = 4)	0.757	96.00 (*n* = 3)	0.042	114.71 (*n* = 3)	0.001
*No*	165.91 (*n* = 67)		501.82 (*n* = 64)		154.15 (*n* = 67)		339.70 (*n* = 64)	
Wound complication (SSO)								
*Yes*	169.12 (*n* = 21)	0.713	463.16 (*n* = 20)	0.469	159.81 (*n* = 20)	0.309	377.56 (*n* = 19)	0.352
*No*	163.79 (*n* = 49)		530.06 (*n* = 47)		147.73 (*n* = 49)		312.82 (*n* = 47)	
Surgical site infection (SSI)								
*Yes*	152.78 (*n* = 6)	0.438	482.95 (*n* = 6)	0.847	142.99 (*n* = 5)	0.553	325.44 (*n* = 5)	0.959
*No*	165.87 (*n* = 66)		507.13 (*n* = 63)		151.80 (*n* = 66)		330.62 (*n* = 63)	
Seroma/hematoma								
*Yes*	171.93 (*n* = 14)	0.605	463.92 (*n* = 13)	0.590	163.21 (*n* = 14)	0.283	410.56 (*n* = 13)	0.237
*No*	163.06 (*n* = 58)		514.58 (*n* = 56)		148.22 (*n* = 57)		311.26 (*n* = 55)	
Reintervention								
*Yes*	144.66 (*n* = 3)	0.201	306.60 (*n* = 3)	0.021	151.10 (*n* = 3)	0.498	258.86 (*n* = 3)	0.193
*No*	166.32 (*n* = 67)		519.63 (*n* = 64)		151.25 (*n* = 66)		334.92 (*n* = 63)	
Readmission								
*Yes*	137.67 (*n* = 2)	0.271	317.43 (*n* = 2)	0.134	144.53 (*n* = 2)	0.453	287.45 (*n* = 2)	0.372
*No*	166.20 (*n* = 68)		516.02 (*n* = 65)		151.44 (*n* = 67)		332.83 (*n* = 64)	
SSI stratified by sex (exploratory post hoc analysis)								
Men								
*SSI Yes*	149.68 (*n* = 5)	0.033	512.73 (*n* = 5)	0.315	139.42 (*n* = 4)	0.107	312.24 (*n* = 4)	0.410
*SSI No*	203.79 (*n* = 22)		702.08 (*n* = 22)		174.79 (*n* = 22)		434.34 (*n* = 20)	
Women								
*SSI Yes*	168.27 (*n* = 1)	0.674	334.07 (*n* = 1)	0.827	157.28 (*n* = 1)	0.72	378.23 (*n* = 1)	0.408
*SSI No*	146.92 (*n* = 44)		402.53 (*n* = 41)		140.3 (*n* = 44)		282.38 (*n* = 44)	

Values are mean (*n*) of preoperative trunk muscle strength parameters in patients with and without each postoperative outcome. p-values result from between-group comparisons (two-sided α = 0.05); missing values were excluded pairwise

Abbreviations: *SSI* Surgical site infection; *PF *Peak force; *RFD* Rate of force development

Surgical outcomes are detailed in Table 4. Length of stay was skewed; the median postoperative stay was 2 days (IQR 1–3; n = 74), with a mean of 4.47 ± 18.19 days. Overall complication rate was 32% (n = 24). Clavien–Dindo grade III complications occurred in 5.6% of patients, while grade IV complications were observed in 1.4%. There were no deaths. Surgical site occurrence (SSO) was present in 32.9% of cases. The most common complications were seroma/hematoma (19.2%), followed by SSI (superficial/deep) in 8.2%. Bleeding occurred in 5.4%; transfusion and reintervention for bleeding were each required in one patient (1.4%). Reintervention rate was 5.5% (mainly wound dehiscence and mesh infection), and readmission rate was 4.1%.

**Table 4:** Postoperative outcomes

Postoperative stay (days) Median (IQR)	2 (1–3)
Complication rate (at least one)	24/75 (32%)
Clavien Dindo (*n* = 75)	
I	87.5%
II	5.6%
III	5.6%
IV	1.4%
V	0%
SSO	26 (32.9)
Seroma + hematoma (%)	14 (19.2)
Hemorrhage (%)	4 (5.4)
SSI (%)	6 (8.2)
Necrosis (%)	1 (1.4)
Wound dehiscence (%)	3 (4.1)
Reintervention rate (%)	4 (5.5)
Readmission rate (%)	3 (4.1)

Values are presented as *n* (%) unless otherwise specified. Postoperative length of stay is reported as median (IQR) due to a skewed distribution (*n* = 74; 1 missing). Where reported, continuous variables are presented as mean ± SD. When reported, continuous variables are presented as mean ± SD

Abbreviations: *LOS* Length of stay; *SSI* Surgical site infection; *SSO* Surgical site occurrence

### Associations between clinical variables (Table 5) and trunk muscle strength (univariable group comparisons)

When examining hernia size, after dichotomizing maximum axial hernia diameter (< 4 cm vs ≥ 4 cm), patients with hernia diameter < 4 cm showed higher lateral peak force (PF45º: 330.07 N vs 178.20 N for ≥ 4 cm; *p* = 0.047). For central muscle performance, no statistically significant difference was observed for RFD90º (369.54 vs 525.36 N/s; *p* = 0.060).

ASA class was associated with higher lateral RFD45º in patients with ASA ≥ 3 compared with ASA < 3 (431.22 vs 288.17 N/s; mean difference ≈ + 143.05 N/s; *p* = 0.021), while differences in PF90º, RFD90º and PF45º did not reach statistical significance. Active smoking was not significantly associated with strength parameters in this cohort.

A history of cardiovascular disease (excluding hypertension) was associated with lower trunk muscle strength, particularly for rate-of-force development: RFD90º (323.09 vs 519.25 N/s; mean difference ≈ − 196.15 N/s; *p* = 0.042), PF45º (117.65 vs 153.72 N; mean difference ≈ − 36.07 N; *p* = 0.021) and RFD45º (162.17 vs 343.58 N/s; mean difference ≈ − 181.41 N/s; *p* = 0.001). PF90º did not reach statistical significance (135.35 vs 166.98 N; *p* = 0.058). These comparisons are unadjusted and may be confounded by factors such as age.

Antithrombotic therapy was associated with higher RFD values (RFD90º: 864.88 vs 464.40 N/s; mean difference ≈ + 400.47 N/s; *p* = 0.011; RFD45º: 590.18 vs 305.09 N/s; mean difference ≈ + 285.10 N/s; *p* = 0.005).

**Table 5:** Association between trunk muscle strength and clinical variables

	PF90 (*N*)	*p*	RFD90 (N/s)	*p*	PF45 (*N*)	*p*	RFD45 (N/s)	*p*
Hernia diameter (CT)								
< *4 cm*	169.49 (*n* = 9)		369.54 (*n* = 9)		330.07 (*n* = 9)		331.55 (*n* = 8)	
> = *4 cm*	164.10 (*n* = 63)	0.793	525.36 (*n* = 60)	0.060	178.20 (*n* = 62)	0.047	330.07 (*n* = 60)	0.981
ASA class								
< *III*	158.41 (*n* = 49)		456.32 (*n* = 47)		149.33 (*n* = 49)		288.17 (*n* = 48)	
> = *III*	178.32 (*n* = 23)	0.094	609.10 (*n* = 22)	0.092	155.29 (*n* = 22)	0.317	431.22 (*n* = 20)	0.021
Diabetes								
*Yes*	172.01 (*n* = 18)		519.29 (*n* = 18)		147.97 (*n* = 18)		307.42 (*n* = 17)	
*No*	162.37 (*n* = 54)	0.278	500.00 (*n* = 51)	0.438	152.26 (*n* = 53)	0.374	337.85 (*n* = 51)	0.343
Active smoking								
*Yes*	168.46 (*n* = 19)		616.02 (*n* = 17)		154.54 (*n* = 19)		403.52 (*n* = 19)	
*No*	163.47 (*n* = 53)	0.378	468.78 (*n* = 52)	0.119	149.95 (*n* = 52)	0.363	301.83 (*n* = 49)	0.079
Cardiovascular history (excluding hypertension)								
*Yes*	135.35 (*n* = 5)		323.09 (*n* = 5)		117.65 (*n* = 5)		162.17 (*n* = 5)	
*No*	166.98 (*n* = 67)	0.058	519.25 (*n* = 64)	0.042	153.72 (*n* = 66)	0.021	343.58 (*n* = 63)	0.001
Respiratory history								
*Yes*	173.93 (*n* = 9)		591.06 (*n* = 8)		152.19 (*n* = 8)		459.44 (*n* = 6)	
*No*	163.47 (*n* = 63)	0.235	493.75 (*n* = 61)	0.243	151.05 (*n* = 63)	0.477	317.74 (*n* = 62)	0.246
Antithrombotic therapy (anticoagulant and/or antiplatelet)								
*Yes*	193.69 (*n* = 7)		864.88 (*n* = 7)		161.84 (*n* = 6)		590.18 (*n* = 6)	
*No*	161.67 (*n* = 65)	0.090	464.40 (*n* = 62)	0.011	150.19 (*n* = 65)	0.288	305.09 (*n* = 62)	0.005

Values are mean (*n*) of preoperative trunk muscle strength parameters. *p*-values result from between-group comparisons (two-sided α = 0.05**)**; missing values were excluded pairwise. Analyses are univariable and exploratory

Abbreviations: *ASA* American Society of Anesthesiologists; *PF* Peak force; *RFD* Rate of force development

### Association between trunk muscle strength and postoperative outcomes (univariable group comparisons)

Bleeding was associated with lower lateral muscle performance: PF45º was 96.00 N in patients with bleeding versus 154.15 N in those without (mean difference ≈ − 58.15 N; *p* = 0.042), and RFD45º was 114.71 N/s versus 339.70 N/s (mean difference ≈ − 224.99 N/s; *p* = 0.001). No meaningful differences were observed for PF90º or RFD90º. Given the low number of bleeding events, these findings are exploratory and may be confounded by other factors (e.g., hernia size).

Reintervention was associated with lower central rate-of-force development: RFD90º was 306.60 N/s in patients requiring reintervention compared with 519.63 N/s in those who did not (mean difference ≈ − 213.03 N/s; *p* = 0.021). Other strength parameters were not significantly different.

Overall SSI was not significantly associated with trunk strength parameters. In an exploratory sex-stratified analysis, men who developed SSI showed lower PF90º compared with men without SSI (149.68 vs 203.79 N; mean difference ≈ − 54.11 N; *p* = 0.033), whereas this pattern was not observed in women; these results should be interpreted cautiously due to small event numbers.

### Correlation analysis

In open repairs, maximum axial hernia diameter was not significantly correlated with trunk strength parameters (Spearman’s ρ: PF90 − 0.094, *p* = 0.488, *n* = 57; PF45 − 0.103, *p* = 0.452, *n* = 56; RFD90 − 0.159, *p* = 0.252, *n* = 54; RFD45 − 0.089, *p* = 0.528, *n* = 53). Age and BMI were not significantly correlated with strength parameters in open repairs (all *p* > 0.05), although an inverse association between age and PF45º did not reach statistical significance (Pearson r = − 0.245, *p* = 0.069, *n* = 56).

### Multivariable analysis

In the adjusted logistic regression model for any 30-day postoperative complication (composite outcome), PF45º was not independently associated with complications (aOR per 10 N: 1.04, 95% CI 0.89–1.22; *p* = 0.590). No covariate reached statistical significance (Table 6). The multivariable model used complete cases (*n* = 56; events = 19).

**Table 6:** Multivariable logistic regression for any 30-day postoperative complication

	aQR	95% CI	p
Age (per 10 years)	1.50	0.89–2.55	0.129
Female sex	0.75	0.19–3.00	0.688
BMI (per 1 kg/m^2^)	0.94	0.83–1.06	0.291
ASA ≥ III	0.93	0.25–3.54	0.921
Hernia diameter (per 1 cm)	1.04	0.83–1.30	0.762
PF45º (per 10 N)	1.04	0.89–1.22	0.590

Outcome: composite 30-day complication (wound complication, bleeding, reintervention, readmission and/or transfusion)

Adjusted odds ratios (aOR) with 95% confidence intervals (CI) from complete-case logistic regression restricted to open repairs (*n* = 56; events = 19). PF45º indicates lateral peak force at 45º

## Discussion

This study supports the concept that incisional hernia management benefits from a dual approach: technical excellence in repair and optimization of patient condition prior to surgery. In this context, objective assessment of abdominal wall function may add clinically relevant information beyond defect morphology alone, potentially contributing to perioperative risk profiling and future prehabilitation strategies. [5, 11–15, 19, 20, 26].

Although incisional hernia is generally reported to be more prevalent in men, our cohort included a higher proportion of women (61.3%), which may be explained by the frequency of prior cesarean sections in our population. [5] Most patients were in the overweight to class I obesity range according to WHO criteria. [6] This aligns with previous evidence linking excess weight to incisional hernia risk and with guideline recommendations emphasizing weight optimization as part of perioperative care pathways. [5, 7].

Regarding comorbidities, diabetes and active smoking remain widely recognized contributors to impaired wound healing and hernia-related risk profiles. [1, 3, 5, 8] In our cohort, diabetes and smoking prevalence were consistent with prior series and with factors highlighted in the EHS guidelines. [5, 8, 31] These considerations reinforce the rationale for targeted optimization strategies, including smoking cessation and metabolic control, as part of comprehensive abdominal wall surgery programs. [5, 12, 19].

From an anatomical standpoint, our cohort represents a selected population of midline incisional hernias with a mean CT-measured diameter of 5.85 cm. Prior studies suggest that larger defects tend to be associated with more impaired abdominal wall function and may be linked to worse postoperative profiles, supporting the clinical relevance of defect size in functional terms [13–15, 31]. Mechanistically, impaired load transmission and reduced mechanical strain may adversely affect fibroblast behavior and collagen remodeling, contributing to fascial weakness and suboptimal tissue repair. [29, 30] Taken together, these observations provide biological plausibility for potential links between abdominal wall function and defect characteristics.

In terms of surgical strategy, retromuscular repair (Rives–Stoppa) was the most frequently employed approach in our series, in accordance with widely adopted recommendations favoring this plane for durable repair and acceptable pain profiles. [5, 32, 33] This cohort included both open and laparoscopic repairs, reflecting real-world individualized decision-making based on hernia characteristics, prior surgery, and surgeon preference. However, the laparoscopic subgroup was small and likely selected; therefore, approach-related comparisons are underpowered and prone to selection bias. Accordingly, primary inferential analyses (correlations and multivariable modelling) were restricted to open repairs, and we avoided interpreting differences between approaches. [5, 9, 10, 34].

Functional assessment of the abdominal wall has been increasingly explored in incisional/ventral hernia populations. Studies have reported that abdominal wall strength is reduced in incisional hernia patients and that increasing defect size relates to worse muscle performance, supporting the plausibility of our research question. [13–15]. Objective testing has been described using portable isometric dynamometry (including strain-gauge devices) and, in other settings, isokinetic systems, with published data supporting feasibility and reproducibility. [24, 25, 35, 37]. Importantly, our study used a standardized strain-gauge protocol, and our findings should be interpreted within this methodological context.

With respect to postoperative outcomes, ventral/incisional hernia repair is known to be susceptible to complications that may contribute to a “vicious cycle” of morbidity, reintervention, and reduced quality of life. [4, 5, 11, 20] In our cohort, univariable analyses suggested associations between lower trunk muscle function and selected early postoperative events. However, these associations do not establish causality or independence and may reflect confounding by age, comorbidity burden, hernia size, and operative complexity. In analyses restricted to open repairs, hernia diameter was not significantly correlated with trunk strength when treated as a continuous variable. In adjusted modelling, PF45º was not independently associated with the composite 30-day complication outcome. Together, these findings suggest that univariable associations should be interpreted cautiously and primarily as hypothesis-generating, particularly given sample size and event counts.

The potential role of structured exercise and rehabilitation in abdominal wall surgery is an area of increasing interest. Functional abdominal wall reconstruction has been associated with improvements in core physiology and quality of life, and prospective data suggest that reconstruction may optimize truncal function compared with baseline. [11, 20]. Provider perspectives also highlight both interest and practical barriers to integrating rehabilitation pathways into routine hernia care [12]. In parallel, emerging evidence and systematic reviews suggest that exercise interventions and core muscle training may influence patient-centered outcomes and can be safe under defined conditions after abdominal surgery, although optimal timing, intensity, and patient selection remain incompletely defined. [19, 26–28, 36, 37]. Future research should clarify whether preoperative functional deficits identify patients most likely to benefit from targeted prehabilitation and whether functional gains translate into improved postoperative outcomes.

This study has certain limitations. Primarily, its single-center design, which may limit external validity. In addition, event numbers for some outcomes were small, increasing imprecision and the risk of type I error in the context of multiple comparisons; therefore, results should be considered exploratory. Third, frailty was not formally assessed using a validated instrument (e.g., handgrip strength or a clinical frailty scale). As trunk muscle function may be influenced by the hernia itself, future studies should incorporate dedicated frailty assessment to improve risk stratification and reduce confounding. Finally, some data on postoperative complications within the first 30 days were collected via patient interviews during follow-up visits, which may have resulted in underreporting minor complications.

Overall, our findings support that abdominal wall function is clinically relevant in incisional hernia patients and warrants further study using standardized objective assessments—such as portable isometric/strain-gauge dynamometry—as part of preoperative characterization. Although adjusted analyses in this cohort did not demonstrate independent prognostic value of PF45º for the composite 30-day complication outcome, larger prospective studies with adequate power, frailty assessment and prespecified models are needed to clarify the role of functional measures and to determine whether targeted perioperative rehabilitation strategies can optimize recovery and reduce complications. [11, 12, 19, 20, 26–28, 35–37].

## Conclusion

Preoperative trunk muscle strength assessment using a standardized strain-gauge protocol is feasible in patients undergoing elective incisional hernia repair. In analyses restricted to open repairs, maximum axial hernia diameter was not significantly correlated with trunk strength when treated as a continuous variable, and in adjusted modelling PF45º was not independently associated with the composite 30-day complication outcome. These findings should be considered exploratory and hypothesis-generating. Multicentre prospective studies with adequate statistical power, incorporating validated frailty assessment and prespecified multivariable models, are warranted before any clinical relevance can be inferred.

## Data Availability

The datasets generated and analyzed during the current study are available from the corresponding author on reasonable request.
